# Autoimmune Regulator Deficiency Results in a Decrease in STAT1 Levels in Human Monocytes

**DOI:** 10.3389/fimmu.2017.00820

**Published:** 2017-07-14

**Authors:** Ofer Zimmerman, Lindsey B. Rosen, Muthulekha Swamydas, Elise M. N. Ferre, Mukil Natarajan, Frank van de Veerdonk, Steven M. Holland, Michail S. Lionakis

**Affiliations:** ^1^Laboratory of Clinical Infectious Diseases, National Institute of Allergy and Immunology, National Institutes of Health, Bethesda, MD, United States; ^2^Department of Internal Medicine, Radboud University Medical Center, Radboud Institute for Molecular Life Sciences (RILMS), Nijmegen, Netherlands

**Keywords:** STAT1, phosphorylation, chronic mucocutaneous candidiasis, *AIRE*, APECED, CD14^+^ monocytes, IFN-γ

## Abstract

Autoimmune polyendocrinopathy-candidiasis-ectodermal dystrophy (APECED) is a rare primary immunodeficiency disorder typically caused by biallelic autoimmune regulator (*AIRE*) mutations that manifests with chronic mucocutaneous candidiasis (CMC) and autoimmunity. Patients with *STAT1* gain-of-function (GOF) mutations also develop CMC and autoimmunity; they exhibit increased STAT1 protein levels at baseline and STAT1 phosphorylation (pSTAT1) upon interferon (IFN)-γ stimulation relative to healthy controls. AIRE interacts functionally with a protein that directly regulates STAT1, namely protein inhibitor of activated STAT1, which inhibits STAT1 activation. Given the common clinical features between patients with *AIRE* and *STAT1* GOF mutations, we sought to determine whether APECED patients also exhibit increased levels of STAT1 protein and phosphorylation in CD14^+^ monocytes. We obtained peripheral blood mononuclear cells from 8 APECED patients and 13 healthy controls and assessed the levels of STAT1 protein and STAT1 tyrosine phosphorylation at rest and following IFN-γ stimulation, as well as the levels of *STAT1* mRNA. The mean STAT1 protein levels in CD14^+^ monocytes exhibited a ~20% significant decrease in APECED patients both at rest and after IFN-γ stimulation relative to that of healthy donors. Similarly, the mean peak value of IFN-γ-induced pSTAT1 level was ~20% significantly lower in APECED patients compared to that in healthy controls. The decrease in STAT1 and peak pSTAT1 in APECED patients was not accompanied by decreased *STAT1* mRNA or anti-IFN-γ autoantibodies; instead, it correlated with the presence of autoantibodies to type I IFN and decreased *AIRE*^−^*^/^*^−^ monocyte surface expression of IFN-γ receptor 2. Our data show that, in contrast to patients with *STAT1* GOF mutations, APECED patients show a moderate but consistent and significant decrease in total STAT1 protein levels, associated with lower peak levels of pSTAT1 molecules after IFN-γ stimulation.

## Introduction

Autoimmune polyendocrinopathy-candidiasis-ectodermal dystrophy (APECED) or autoimmune polyglandular syndrome type-1 (APS-1; OMIM 240300) is a monogenic disorder caused by biallelic mutations in the autoimmune regulator (*AIRE*) gene, a thymus-enriched transcription regulator that promotes central immune tolerance *via* the expression of peripheral tissue self-antigens in medullary thymic epithelial cells (1, 2). Additional AIRE functions have recently been proposed to also contribute to immunological tolerance (3–6). In addition, heterozygous dominant-negative *AIRE* mutations in the plant homeodomain 1 domain have also been described, associated with organ-specific APECED-associated autoimmune manifestations and/or chronic mucocutaneous candidiasis (CMC) (7–9). APECED patients manifest with a characteristic constellation of CMC and autoimmunity that involves both endocrine and non-endocrine tissues (10–12). In fact, APECED is the only CMC-associated primary immunodeficiency disorder in which CMC is the sole consistent infectious disease phenotype. In addition to the breakdown in mechanisms of T-cell tolerance, AIRE-deficient patients also have high titers of neutralizing autoantibodies against Th17 cytokines and tissue-specific autoantigens, which have been shown to correlate with the development of CMC and organ-specific autoimmune manifestations, respectively (13–15). In addition, APECED patients exhibit a decreased frequency of peripheral blood IL-17-producing CD4^+^ T cells following PMA/ionomycin stimulation [(3, 15), Lionakis, unpublished observations].

Heterozygous *STAT1* gain-of-function (GOF) mutations were initially implicated in causing autosomal-dominant CMC (16, 17) but have thereafter also been associated with the development of autoimmunity that can involve endocrine and non-endocrine tissues (16–19); beyond these common clinical features between APECED and *STAT1* GOF mutations, patients with *STAT1* GOF mutations also develop a broad-spectrum of infectious, inflammatory, and vascular manifestations not seen in APECED (19). These *STAT1* mutations are considered GOF because of enhanced phosphorylated STAT1 molecules upon interferon (IFN)-γ stimulation (17, 20). Impaired production of Th17 cytokines by T-cells has been implicated in the pathogenesis of CMC in these patients (17, 19, 20).

Due to the overlap in CMC and other clinical features between patients with APECED and *STAT1* GOF mutations, and because AIRE interacts functionally with a protein inhibitor of activated STAT1 (PIAS1), which inhibits STAT1 activation (21, 22), we aimed to study STAT1 protein level and phosphorylation upon IFN-γ stimulation in patients with *AIRE* mutations and determine whether human AIRE deficiency phenocopies the cell-intrinsic enhanced STAT1 levels seen in patients with *STAT1* GOF mutations.

## Materials and Methods

### Study Participants

Eight APECED patients were enrolled (2015–2017) on a NIAID IRB-approved protocol and provided written informed consent. All eight had a clinical diagnosis of APECED based on the development of any two manifestations within the classic triad of CMC, hypoparathyroidism, and adrenal insufficiency. The most common clinical manifestations among the eight APECED patients included CMC (88%), hypoparathyroidism (100%), adrenal insufficiency (88%), and enamel hypoplasia (100%). The full list and frequencies of clinical manifestations of the eight APECED patients are outlined in Table 1. The most common *AIRE* mutant allele in these eight patients was c.967_979del13 (60%), followed by c.769C>T (13%). Two patients were compound heterozygous for c.967_979del13 and c.769C>T, while the remaining six patients had six different *AIRE* genotypes. Samples from a patient carrying the c.1057G>A E353K *STAT1* GOF mutation and a patient with the autosomal dominant form of IFN-γ receptor 1 deficiency carrying the 818del4 mutation were also collected under a NIAID IRB-approved protocol and provided written informed consent. Healthy volunteer blood samples from 13 individuals were obtained for STAT1 protein evaluation and from 10 individuals for *STAT1* mRNA level determination under IRB-approved protocols through the Department of Transfusion Medicine, at the NIH Clinical Center. The study was conducted in accordance with the Helsinki Declaration.

**Table 1:** Clinical manifestations of the eight autoimmune polyendocrinopathy-candidiasis-ectodermal dystrophy patients included in this study.

Manifestation^a^	Number of affected patients	% of affected patients
Adrenal insufficiency	7	88
Asplenia	1	13
Autoimmune hepatitis	2	25
Alopecia	1	13
B12 deficiency	3	38
Chronic mucocutaneous candidiasis	7	88
Enamel hypoplasia	8	100
Gastritis	3	38
Gonadal failure	3	38
Early-onset hypertension	4	50
Hypoparathyroidism	8	100
Hypothyroidism	3	38
Intestinal dysfunction	6	75
Keratoconjunctivitis	1	13
Nail dystrophy	1	13
Tubulointerstitial nephritis	1	13
Pneumonitis	2	25
Sjogren’s-like syndrome	3	38
Urticarial eruption	3	38
Vitiligo	1	13

*^a^The manifestations are presented in alphabetical order*.

### Peripheral Blood Mononuclear Cells (PBMC) Isolation and Intracellular Staining for STAT1 and pSTAT1

STAT1 protein and pSTAT1 levels were examined using flow cytometry in paired APECED patients and healthy control individuals in seven independent experiments at rest and up to 60 min after IFN-γ stimulation. Each patient and healthy donor was evaluated only once. In six of the seven independent experiments, a single APECED patient was evaluated along with two accompanying healthy donors, and in one experiment two APECED patients were evaluated together with one accompanying healthy donor.

PBMC were isolated by density-gradient centrifugation using lymphocyte separation media (Lonza) and resuspended in RPMI culture media (Gibco), supplemented with pyruvate (100 mM, Sigma Aldrich), glutamate (200 mM, Life Technologies), penicillin/streptomycin (100 U/100 μg/ml, Life Technologies), 10% fetal bovine serum (Serum Source International) and HEPES (20 mM, General Electric).

Intracellular phosphorylated STAT1 (pSTAT1) and total STAT1 were determined by FACS analysis, as previously described (18, 23). Freshly isolated PBMC were resuspended at 10^6^ cells per 100 µl in RPMI and were serum starved for 30 min, in polystyrene round-bottom tubes (Becton Dickinson Falcon). Cells were then incubated with FITC-conjugated anti-human CD14 (Becton Dickinson cat# 555397). Cells were then stimulated with IFN-γ (800 U/ml) for 15, 30, or 60 min at 37°C, fixed with 2% Paraformaldehyde (Electron Microscopy Sciences) at 37°C for 10 min, permeabilized with 100% methanol in dark on ice for 30 min, washed with PBS/2%FBS, and incubated for 1 h in the dark at 4°C with combinations of PerCP–Cy5.5-conjugated anti-human pSTAT1 (Y701) (Becton Dickinson cat# 560113) and Alexa Fluor 647-conjugated anti-human N-terminus STAT1 (Becton Dickinson cat# 558560) with Fix and Perm Medium B (Life technologies). Alexa Fluor 647-conjugated IgG1 isotype control (Becton Dickinson cat# 557783) was used. Baseline pSTAT1 levels were used as a control for the specificity of the PerCP–Cy5.5-conjugated anti-human pSTAT1 antibody, by comparing between pSTAT1 levels as expressed in geometric mean of fluorescence-at rest and after stimulation. Samples were washed once with PBS/2%FBS and resuspended in 1% Paraformaldehyde. All data were collected with LSR Fortessa or LSRII (Becton Dickinson) and analyzed with FlowJo software (Treestar, Ashland, OR, USA).

### IFN-α, IFN-ω, and IFN-γ Autoantibody Detection

A particle-based multiplex assay was used to detect IFN-α, IFN-ω, and IFN-γ autoantibodies in the serum or plasma samples from the eight APECED patients and compared with healthy control subjects (*n* = 100) enrolled through the NIH Blood Bank, as previously described (24).

### IFN-γ Receptors 1 and 2 Expression on Monocytes

Frozen PBMC from eight APECED patients and eight healthy donors were used for measuring IFN-γ receptors 1 and 2 levels on CD14^+^ monocytes. Cells were resuspended at 10^6^ cells per 100 µl in PBS and incubated with LIVE/DEAD^®^ Fixable Aqua Dead Cell Stain Kit (ThermoFisher) in 4°C followed by staining with FITC-conjugated anti-human CD14 (Becton Dickinson cat# 555397), PE-conjugated anti CD119 (IFN-γ receptor 1; IFN-γR1; Becton Dickinson cat# 558937), or APC-conjugated anti-IFN-γ receptor 2 (IFN-γR2; R&D cat# FAB773A) for 30 min. PE-conjugated IgG2b κ isotype control (Cat# 555058) and APC-conjugated IgG isotype control (R&D cat# IC108A) were used for control staining. Cells were then washed with PBS/2%FBS and fixed with 1% PFA. Data were collected with LSRII (Becton Dickinson) and analyzed with FlowJo software (Treestar, Ashland, OR, USA). The geometric mean fluorescence intensity on monocytes for each receptor was calculated after subtracting the geometric mean fluorescence intensity of the corresponding isotype control staining.

### STAT1 mRNA Expression Determination

Frozen PBMC from the 8 APECED patients and 10 healthy donors were used for measuring *STAT1* mRNA expression by quantitative PCR (qPCR). To determine the percent of live CD14^+^ monocytes within PBMC, an aliquot of the PBMC was incubated with LIVE/DEAD^®^ Fixable Violet Dead Cell Stain Kit (ThermoFisher) in 4°C followed by staining with FITC-conjugated anti-human CD14 (Becton Dickinson cat# 555397). Data were collected with LSRII (Becton Dickinson) and analyzed with FlowJo software (Treestar, Ashland, OR, USA). Among the live PBMC, the mean percentage of CD14^+^ monocytes was similar in the patient and healthy donor groups: 7.2 ± 0.8 vs. 7.7 ± 1.7, respectively (*p* = 0.75). For mRNA extraction, the RNeasy kit (Qiagen) was used, according to the manufacturer’s instructions. To convert mRNA to cDNA, the high-capacity cDNA reverse transcription kit (Applied Biosystems) was used. qPCR was then performed with TaqMan detection (TaqMan^®^ Universal Master Mix II, with UNG; ThermoFisher), using the 7,500 real-time PCR system (Applied Biosystems) and predesigned primer and probe mixes for glyceraldehyde-3-phosphate dehydrogenase (GAPDH; ThermoFisher) or STAT1 (ThermoFisher). All qPCR assays were performed in duplicate and results were normalized to GAPDH transcript levels using the threshold cycle (Ct) method.

### AIRE Sequencing

Genomic DNA was extracted from whole blood, amplified and sequenced for *AIRE* exons and flanking splice sites as previously described (25).

### Statistical Analysis

The geometric mean of fluorescence for pSTAT1 or STAT1 protein levels was calculated using the FlowJo software and the values obtained from APECED patients were normalized to the values obtained from the same-day healthy control samples. All results were expressed as mean ± SEM unless otherwise indicated. Statistical analyses were performed by Student’s *t*-test or Mann–Whitney U test, where appropriate, using the GraphPad Prism 7 software (La Jolla, CA, USA). A *p* value of less than 0.05 was considered significant.

## Results

**Figure 1:**
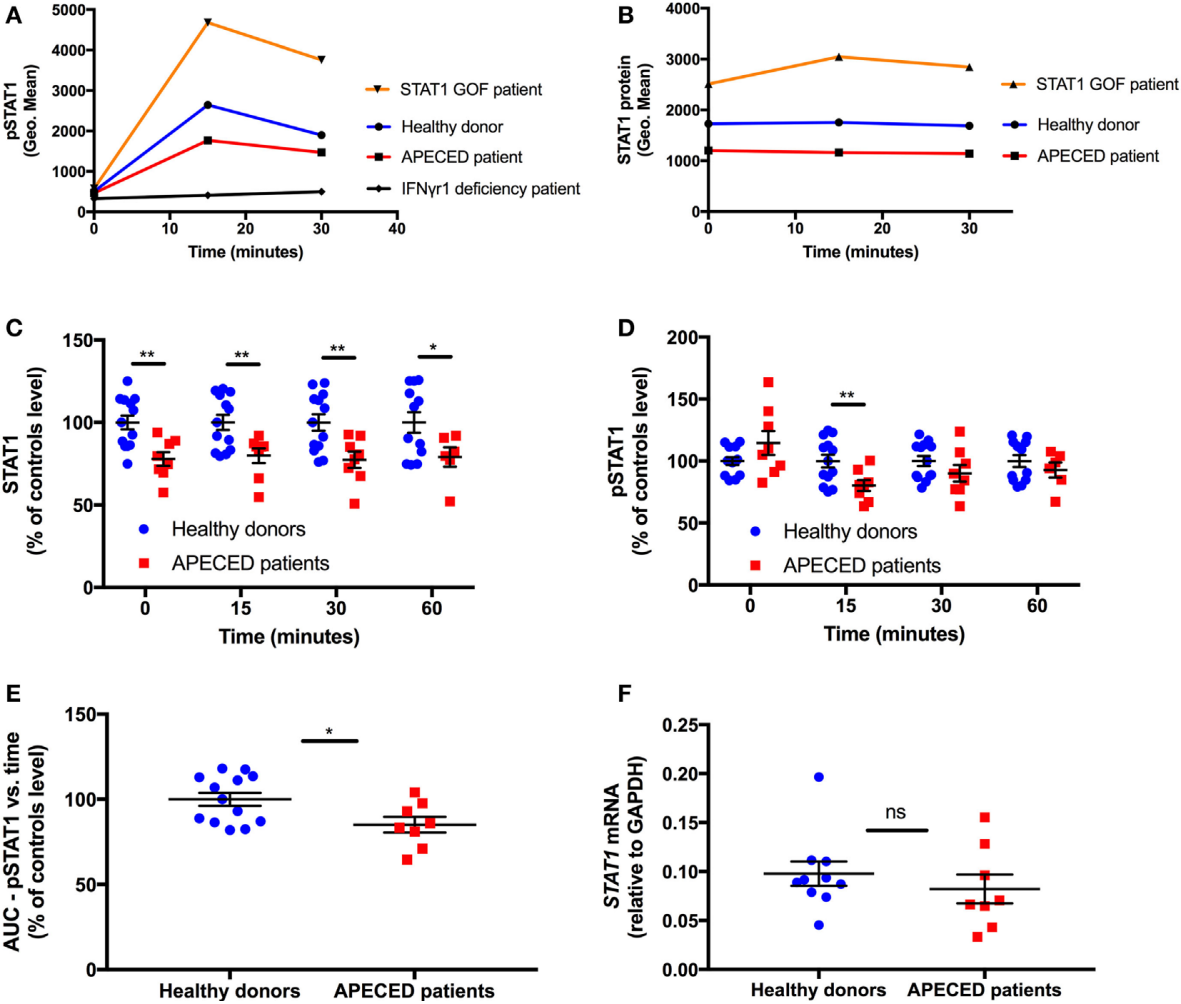
Autoimmune regulator deficiency results in a decrease in STAT1 protein levels in human monocytes. **(A)** Representative depiction of pSTAT1 level at rest and up to 30 min after interferon (IFN)-γ stimulation in CD14^+^ cells of a *STAT1* gain-of-function (GOF) patient (orange), an autoimmune polyendocrinopathy-candidiasis-ectodermal dystrophy (APECED) patient (red), a patient with the autosomal dominant form of IFN-γR1 deficiency (black) and a healthy donor (blue) **(B)** Representative depiction of STAT1 protein level at rest and up to 30 min after IFN-γ stimulation in CD14^+^ cells of a *STAT1* GOF patient (orange), an APECED patient (red), and a healthy donor (blue). Protein and phosphorylation levels are expressed in geometric mean of fluorescence (Geo. Mean), as measured by flow cytometry. STAT1 total protein **(C)** and pSTAT1 **(D)** levels in CD14^+^ cells of APECED patients (*n* = 8; red dots) and healthy donors (*n* = 13; blue dots) at rest (time 0) and up to 60 min after IFN-γ stimulation. Total protein and phosphorylation levels are expressed in % of the same-day control average values, for each time point—0, 15, 30, and 60 min, separately. **(E)** Area under the curve of CD14^+^ cells STAT1 phosphorylation vs. time in APECED patients (*n* = 8; red dots) and healthy donors (*n* = 13; blue dots). **(F)** *STAT1* mRNA level, relative to glyceraldehyde-3-phosphate dehydrogenase, in peripheral blood mononuclear cells of healthy donors (*n* = 10) and APECED patients (*n* = 8) at rest. ns, not significant. *p < 0.05; **p < 0.01, by *t-*test. Quantitative data represent mean ± SEM.

We enrolled eight APECED patients from eight non-consanguineous families with clinical and genetic diagnosis of APECED. Two (25%) were male and six (75%) were female. The mean patient age was 22 years (range, 9–56 years); 3 (38%) were children, with a mean age of 11 years. Thirteen healthy donors were enrolled for same-day harvesting and comparative analyses of STAT1 and pSTAT1 protein levels. A *STAT1* GOF patient was tested as control for enhanced STAT1 protein and pSTAT1 levels [patient 1 in Ref. (20)], and a patient with the autosomal dominant form of IFN-γR1 deficiency [patient described in the case report in Ref. (26)] was tested as control for absent IFN-γ-induced pSTAT1 stimulation (Figures 1A,B).

We examined STAT1 protein and pSTAT1 levels in paired APECED patients and healthy control individuals, at rest and up to 60 min after IFN-γ stimulation. We focused on IFN-γ because it induces STAT1 phosphorylation and homo-dimerization without the involvement of other STAT molecules as in the case of STAT1–STAT2 heterodimer formation induced by IFN-α stimulation (27, 28). We also focused on CD14^+^ monocytes because of their relatively high levels of IFN-γ receptors 1 and 2, which allows for detection of rapid activation of STAT1 (29).

In all the eight tested APECED patients, STAT1 protein levels were lower than the same-day healthy donors mean levels (Figure 1C). The eight patients’ mean CD14^+^ monocyte STAT1 protein level at rest was ~80% of that observed in healthy donors (*p* = 0.003). After IFN-γ stimulation, the significant decrease in CD14^+^ monocyte mean STAT1 protein level of APECED patients persisted at all examined time-points throughout the 60 min of the experiment (Figure 1C).

Consistent with previous reports (23), we found that pSTAT1 induction peaked 15 min after IFN-γ stimulation and started to decline toward baseline at 30 min after stimulation in all 13 healthy donor monocytes (Figure 1A). Similar kinetics of pSTAT1 induction peak and decline were observed in all eight APECED patients (Figure 1A). APECED patient monocytes’ mean pSTAT1 levels at rest were not significantly different compared to healthy donor levels at rest (108.7 ± 9.2% vs. 100 ± 2.83%, respectively, *p* = 0.29; Figure 1D). However, the mean peak pSTAT1 level of the APECED patients was 20% decreased compared to that seen in healthy donors at 15 min (80 ± 4.5% vs. 100 ± 4.5%, respectively. *p* = 0.008). Thereafter, at 30 min after IFN-γ stimulation, the patients’ mean pSTAT1 level was lower than healthy donors (87 ± 5.1% vs. 100 ± 3.9%, respectively), and the difference was close to statistical significance (*p* = 0.06), whereas at 60 min after IFN-γ stimulation the differences in pSTAT1 levels between the patients and healthy donors groups were not significant (91 ± 5.5% vs. 100 ± 4.3%, respectively, *p* = 0.25; Figure 1D). We also calculated the area under the curve (AUC) of STAT1 phosphorylation (expressed as geometric mean of fluorescence) vs. time (expressed as minutes) for up to 60 min after IFN-γ stimulation in both groups and compared patient AUC level to the same-day healthy donor average. Consistent with the mean peak pSTAT1 values, APECED patients mean AUC levels were ~15% decreased relative to those seen in healthy controls (*p* = 0.025, Figure 1E). Because Th17 cells are decreased in peripheral blood of APECED [(3, 15), Lionakis, unpublished observations] and STAT1 GOF patients (17, 19, 20), our findings collectively indicate that the Th17 frequency decrease in AIRE deficiency is not caused by a STAT1 GOF state in peripheral blood monocytes.

Because among the eight APECED patients, seven different *AIRE* genotypes were observed, reflecting the greater genetic diversity of APECED in North America (25), we were not able to examine whether there is a correlation between specific *AIRE* mutations and the degree of pSTAT1 and total STAT1 levels in AIRE-deficient monocytes.

Given the decreased STAT1 protein levels seen in APECED patients, we asked whether *STAT1* is also decreased at the mRNA level in APECED patients. Thus, we compared *STAT1* mRNA levels by qPCR in PBMC of 8 APECED patients and 10 healthy donors and found them to be similar (*p* = 0.32) (Figure 1F).

**Figure 2:**
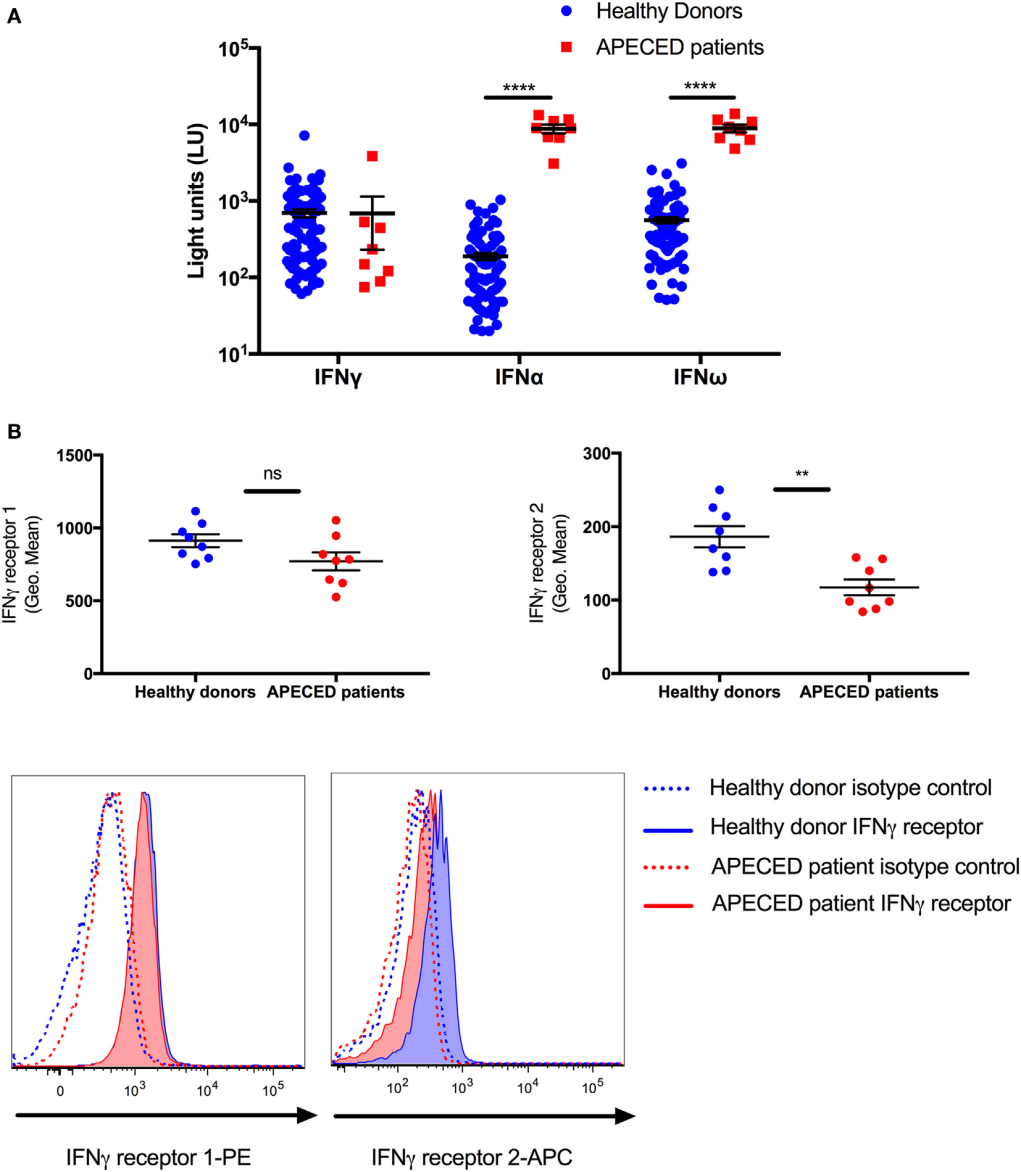
The decrease in STAT1 protein and pSTAT1 levels seen in autoimmune polyendocrinopathy-candidiasis-ectodermal dystrophy (APECED) patients correlates with lower monocyte interferon (IFN)-γR2 receptor expression and the presence of type I IFN autoantibodies. **(A)** Sera from 8 APECED patients and 100 healthy donors were evaluated for the presence of autoantibodies against IFN-γ, IFN-α, and IFN-ω. Shown is autoantibody immunoreactivity against the indicated cytokines expressed as fluorescence intensity using a particle-based multiplex assay. **(B)** IFN-γ receptor 1 and 2 levels were measured in CD14^+^ monocytes of healthy donors (*n* = 8) and APECED patients (*n* = 8). Summary data on mean fluorescence intensity and representative histogram FACS plots are shown. **p < 0.01; ****p < 0.0001, by Mann–Whitney U test **(A)** or *t*-test **(B)**. Quantitative data represent median ± SEM.

Because APECED patients are known to have autoantibodies against cytokines, we examined whether autoantibodies to type I IFNs or IFN-γ were present in the serum of the eight APECED patients, which could potentially affect the levels of pSTAT1 in the patients group, after IFN-γ stimulation. All eight tested patients had autoantibodies to IFN-α and IFN-ω, while no patient had autoantibodies to IFN-γ (Figure 2A).

Given the decreased peak pSTAT1 level after IFN-γ stimulation in AIRE-deficient monocytes, we also asked whether the surface expression of IFN-γ receptors 1 and 2 is decreased in the monocytes of the eight APECED patients using flow cytometry. We found no difference in the expression of IFN-γR1 between healthy donor and APECED monocytes but a significant ~40% decrease in surface expression of IFN-γR2 in patient monocytes (mean fluorescence intensity 186.4 ± 14.61 vs. 117.3 ± 10.68, *p* = 0.002, respectively, Figure 2B).

## Discussion

Because of the similarities in clinical presentation of CMC and autoimmunity and decreased peripheral blood Th17 frequencies between patients with APECED and *STAT1* GOF mutations, our study examined STAT1 protein and phosphorylation levels in patients with APECED and shows that APECED patient monocytes do not share the same cell-intrinsic increase in STAT1 protein and phosphorylation levels as cells from patients with *STAT1* GOF mutations. In fact, our findings reveal a moderate but consistent and significant decrease in STAT1 protein and phosphorylation levels in APECED CD14^+^ monocytes at rest and after IFN-γ stimulation.

Until recently, it had been postulated that impaired nuclear dephosphorylation is the underlying cause of increase in pSTAT1 levels in patients with *STAT1* GOF mutations (17, 19, 20). Nonetheless, recent reports have described normal dephosphorylation rates in some patients with a *STAT1* GOF mutations; for example, Sobh et al. (30), Meesilpavikkai et al. (31), and Weinacht et al. (32) described three new *STAT1* GOF mutations in the SH2 (p.H629Y and p.V653I) and linker (p.E545K) domains with enhanced pSTAT1 levels but normal dephosphorylation. In addition, Tabellini and colleagues recently reported high levels of STAT1 protein in NK cells from seven patients carrying five different CC or DNA binding (DB) domain *STAT1* GOF mutations (23). Similarly, high STAT1 protein levels were found in CD14^+^ monocytes and CD3^+^ T cells of 12 patients with 10 different CC, DB, or SH2 domain *STAT1* GOF mutations (Zimmerman and Holland, submitted manuscript). After controlling the *STAT1* GOF patients’ pSTAT1 levels for the corresponding total STAT1 protein levels, STAT1 phosphorylation levels were normal (Zimmerman and Holland, submitted manuscript). These findings collectively support the hypothesis that high resting and IFN-γ-stimulated total STAT1 protein levels may serve as a background against which high pSTAT1 levels occur in some patients with *STAT1* GOF mutations. Alternatively, the pSTAT1 levels may not directly correlate with total STAT1 levels, as indicated in other reports (33). Future studies using more STAT1 GOF and STAT1 loss-of-function (LOF) patients will be needed to determine the temporal dynamics and correlation of total and pSTAT1 molecules.

In our study, based on the presence of CMC and other shared clinical features between APECED and *STAT1* GOF mutation patients, we tested the hypothesis that AIRE deficiency enhances STAT1 cellular responses as a result of enhanced STAT1 protein and phosphorylation levels. Instead, we found a ~20% decrease in STAT1 protein and peak pSTAT1 levels in APECED patients; the decrease in total STAT1 levels could indicate that the decreased STAT1 protein level may be a determining factor for the decreased peak STAT1 phosphorylation levels observed in APECED CD14^+^ monocytes, although it could be accounted for by alternative factors that remain to be elucidated.

The molecular mechanism behind our finding is currently unknown. One possible explanation could have been that AIRE is involved in the regulation of STAT1 transcription; however, we found no significant difference in *STAT1* mRNA levels between healthy donor and APECED PMBCs. Alternatively, AIRE may regulate STAT1 post-transcriptionally at the level of degradation or SUMOylation or other process directly or indirectly *via* known (e.g., PIAS1) or yet-unknown protein partners (21, 22). Alternatively, APECED patient autoantibodies against type I IFNs, which have been shown to exhibit the ability to neutralize and block IFN-α activity, STAT1 phosphorylation, and the expression of interferon-stimulated genes (34, 35), may adversely affect tonic STAT1 expression in blood monocytes. The decrease in IFN-γR2 expression on CD14^+^ monocytes may be a contributor to the decreased peak pSTAT1 levels in APECED patients’ cells. Future studies will be required to further examine this hypothesis as well as molecules and functional responses downstream of STAT1.

Impaired STAT1-dependent responses in patients with LOF *STAT1* mutations or in patients with defects in the IFN-γ receptor signaling axis, underlie Mendelian susceptibility to mycobacterial disease (36). The defect in STAT1 levels post-IFN-γ stimulation that we observed in our APECED patients is modest relative to the complete absence of STAT1 phosphorylation following IFN-γ stimulation in patients with IFN-γ receptor deficiency [(23); Figure 1A]. Therefore, significant residual STAT1 signaling is functional in APECED patients to prevent the development of mycobacterial infections in these patients. Whether the decrease in STAT1 protein and phosphorylation that we identified is a contributing factor to the development of viral infections in some APECED patients [(37); Lionakis, unpublished observations] remains to be elucidated. However, the decrease in STAT1 levels is unlikely to contribute to the pathogenesis of CMC as patients with defects in the IFN-γ receptor signaling axis do not have Th17 defects and do not develop CMC (36). Hence, at this point, the biological and clinical implications of the decreased STAT1 levels in patients’ monocytes are unclear.

In summary, we describe an association between AIRE deficiency and a decrease in STAT1 protein level in primary human monocytes that is not accompanied by decreased *STAT1* mRNA levels but correlates with the presence of type I IFN autoantibodies and decreased monocyte surface expression of IFN-γR2. The mechanism behind this finding and its clinical and biological implications in APECED patients require further investigation.

## Ethics Statement

Eight APECED patients were enrolled (2015–2017) on a NIAID IRB-approved protocol and provided written informed consent. Samples from a patient carrying the c.1057G>A E353K STAT1 GOF mutation and a patient with the autosomal dominant form of IFN-γ receptor 1 deficiency carrying the 818del4 mutation were also collected under a NIAID IRB-approved protocol and provided written informed consent. Healthy volunteer blood samples from 13 individuals were obtained for STAT1 protein evaluation and from 10 individuals for STAT1 mRNA level determination under IRB-approved protocols through the Department of Transfusion Medicine, at the NIH Clinical Center. The study was conducted in accordance with the Helsinki Declaration.

## Author Contributions

ML conceived the project and contributed to the design and supervision of the experiments. OZ and ML wrote the manuscript. OZ, LR, and MS conducted experiments and generated manuscript figures. OZ, LR, MN, FV, and ML contributed to the interpretation of the data. EF contributed to healthy donor enrollment. SH provided patient care contributed patients’ samples critical for the study execution.

## Conflict of Interest Statement

The authors declare that the research was conducted in the absence of any commercial or financial relationships that could be construed as a potential conflict of interest.

## Abbreviations

APECED, autoimmune polyendocrinopathy-candidiasis-ectodermal dystrophy; APS-1, autoimmune polyglandular syndrome type-1; CC, coiled-coil; CMC, chronic mucocutaneous candidiasis; DB, DNA binding; GOF, gain-of-function; LOF, loss-of-function; PBMC, peripheral blood mononuclear cells; STAT, signal transducer and activator of transcription.
